# Neural tissue-engineered prevascularization *in vivo* enhances peripheral neuroregeneration via rapid vascular inosculation

**DOI:** 10.1016/j.mtbio.2023.100718

**Published:** 2023-06-30

**Authors:** Hongkui Wang, Ping Zhang, Panjian Lu, Xiaodong Cai, Gang Wang, Xi Xu, Ying Liu, Tianyi Huang, Meiyuan Li, Tianmei Qian, Hui Zhu, Chengbin Xue

**Affiliations:** a Key Laboratory of Neuroregeneration of Jiangsu and Ministry of Education, Co-innovation Center of Neuroregeneration, NMPA Key Laboratory for Research and Evaluation of Tissue Engineering Technology Products, Jiangsu Clinical Medicine Center of Tissue Engineering and Nerve Injury Repair, Research Center of Clinical Medicine, Affiliated Hospital of Nantong University, Nantong University, Nantong, JS, 226001, PR China; b Department of Rehabilitation Medicine, Affiliated Hospital of Nantong University, Nantong, JS, 226001, PR China; c Department of Pathology, Affiliated Hospital of Nantong University, Nantong, JS, 226001, PR China; d Medical School of Nantong University, Nantong, JS, 226001, PR China

**Keywords:** Prevascularization, Neuroregeneration, Vascular inosculation, Tissue engineered nerve graft, Peripheral nerve injury

## Abstract

Neural tissue engineering techniques typically face a significant challenge, simulating complex natural vascular systems that hinder the clinical application of tissue-engineered nerve grafts (TENGs). Here, we report a subcutaneously pre-vascularized TENG consisting of a vascular endothelial growth factor-induced host vascular network, chitosan nerve conduit, and inserted silk fibroin fibers. Contrast agent perfusion, tissue clearing, microCT scan, and blood vessel 3D reconstruction were carried out continuously to prove whether the regenerated blood vessels were functional. Moreover, histological and electrophysiological evaluations were also applied to investigate the efficacy of repairing peripheral nerve defects with pre-vascularized TENG. Rapid vascular inosculation of TENG pre-vascularized blood vessels with the host vascular system was observed at 4 ​d bridging the 10 ​mm sciatic nerve defect in rats. Transplantation of pre-vascularized TENG *in vivo* suppressed proliferation of vascular endothelial cells (VECs) while promoting their migration within 14 ​d post bridging surgery. More importantly, the early vascularization of TENG drives axonal regrowth by facilitating bidirectional migration of Schwann cells (SCs) and the bands of Büngner formation. This pre-vascularized TENG increased remyelination, promoted recovery of electrophysiological function, and prevented atrophy of the target muscles when observed 12 weeks post neural transplantation. The neural tissue-engineered pre-vascularization technique provides a potential approach to discover an individualized TENG and explore the innovative neural regenerative process.

## Highlights

• An individualized TENG is developed for repairing sciatic nerve defect.
• Rapid vascular inosculation via neural tissue-engineered prevascularization *in vivo* enhances peripheral neuroregeneration.
• Pre-vascularization *in vivo* suppress proliferation and promote migration of vascular endothelial cell.
• Early vascularization of TENG drive axonal regrowth by facilitating bidirectional migration of Schwann cells.
• Pre-vascularized TENG increase remyelination and restoration of neural function.

## Graphical abstract

**Schematic illustration of an *in vivo* prevascularization of tissue-engineered nerve graft for the sciatic nerve repair.** A subcutaneously pre-vascularized TENG consisting of a vascular endothelial growth factor (VEGF) induced host vascular network, chitosan nerve conduit, and inserted silk fibroin fibers. Rapid vascular inosculation occurred with the host vascular system to repair the 10 ​mm sciatic nerve defect in rat.

**Figure:**
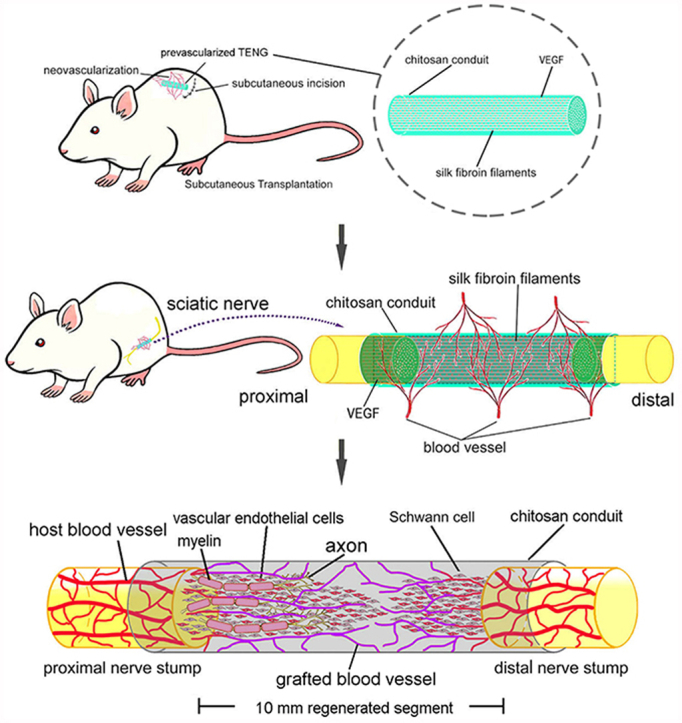

## 1 Introduction

Despite significant progress in neural tissue engineering, extra efforts have been made toward repairing and reconstructing long-distance nerve-trunk defects [1]. For human peripheral nerve injuries, axons typically regenerate much longer distances than rodents, and target muscles lose innervation for extended amounts of time [2]. The vascularization is essential to ensure the survival and regeneration of the tissue-engineered nerve grafts (TENGs) that is closely related to the repair effect of peripheral nerves [3]. Since the blood vessels may direct the migrating cords of Schwann cells (SCs), interfering the neural angiogenesis *in vivo* or compromising directionality of SCs will lead to inferior nerve repair [4]. Promoting the early vascularization of TENGs is particularly important. However, simulating the complex natural vascular system *in vitro* faces enormous challenges. The research on TENGs is not limited to biological materials, seed cells, cytokines, and other active ingredients. Further research on biomaterials and engineering technology is urgently needed [5].

Currently, there is no off-the-shelf biomaterials can efficiently induce angiogenesis, and proangiogenic drugs typically suffer from their abnormal angiogenesis and potential cancer risk [6]. The tissue engineered prevascularization techniques (as coculture, cell sheet technology, spheroids, three-dimensional (3D) bioprinting, and microfluidic technology [3,[7], [8], [9]]) mainly base on: ①vascular endothelial cells (VECs) [10,11] (or VECs differentiated from stem cell [12,13]) or various types of cells as VECs, fibroblasts, and SCs [11], and then transplanting vascularized engineered grafts enter the body and coincide with the angiogenesis of the host; ② growth factors or cytokines that promote angiogenesis include VEGF, bFGF, HGF, etc. [[14], [15], [16]]. In addition, PDGF, TGF-β, Ang, etc. [17,18] can indirectly promote the regeneration of VECs and accelerate the process of vascularization; ③biological scaffold materials, which are consist of natural biologically derived vascularized scaffold materials (such as decellularized matrix [19], chitosan [20], silk fibroin [21], fibrin [22], etc.) and artificially synthesized vascularized scaffolds materials (such as polycaprolactone [23], polyethylene glycol [24], etc.).

Two principal vascularization strategies in tissue engineering are angiogenesis and inosculation. The angiogenesis approach is characterized by the in growth of vascular sprouts from the host microvasculature into an implanted tissue construct, which form a new microvascular network. Thus, rapid blood perfusion is difficult to achieve. In case of the inosculation approach, a preformed microvascular network is created within a graft before the implantation. This bears the advantage that the preformed micro-vessels simply have to develop inosculation to the host microvasculature to get fully blood-perfused within a short period of time that may depend on the emerging internal or external inosculation [25].

Over past twenty years, we contributed to the development of desirable biomaterials as chitosan [[26], [27], [28], [29]], silk fibroin [30,31], and extracellular matrix (ECM) for fabricating neural scaffolds to repair peripheral nerve defects [1,32,33] Among them, chitosan based TENG has accomplished the industry translation from basic research to clinical medical device in China (Eton-Biotech). The wall of the chitosan nerve conduit we used owns a microporous structure that allows blood vessels, nutrients, oxygen, and growth factors to penetrate. The microporous structure can expel debris and other wastes in the early stage of an inflammatory response, preserve neurotrophic growth factors, and prevent cell infiltration in the process of axon regeneration [32]. The bioactive materials may guide the host cells and affect the microenvironment to promote *in situ* angiogenesis [6].

**Fig. 1:**
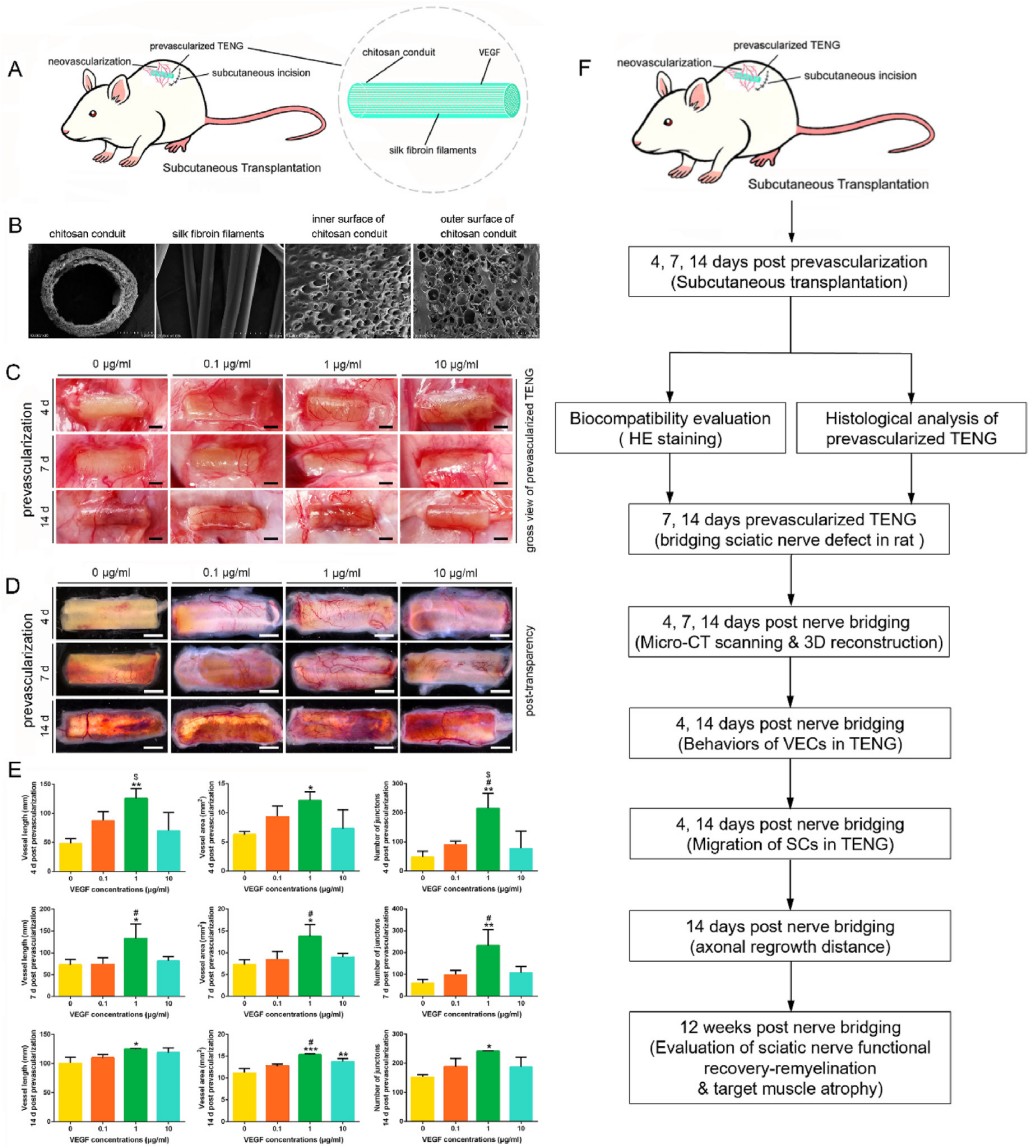
Gross view of the prevascularized TENGs after subcutaneous prevascularization. Compared with other concentrations, the 1 ​μg/ml VEGF better promoted the neovascularization of TENGs under the condition of subcutaneous implantation *in vivo* at different time points. (A) A schematic diagram of the TENGs prevascularization approach. (B) Scanning electron microscope picture of cross section of chitosan conduit, silk fibroin filaments, inner surface and outer surface of chitosan conduit. The wall of nerve conduit displayed porous structure. Scale bar, 1 ​mm, 50 ​μm, 50 ​μm, 200 ​μm. (C)The appearance of prevascularized TENGs in a suit following subcutaneous implantation at 4 ​d, 7 ​d, and 14 ​d. The different concentrations of VEGF combined with chitosan nerve conduits were designed: 0.1 ​μg/ml, 1 ​μg/ml, and 10 ​μg/ml. Scale bar, 2000 ​μm. (D) The appearance of TENGs post-transparency with different VEGF concentrations at different prevascularization times. Scale bar, 3000 ​μm. (E) Histograms of blood vessel statistics of the TENGs post-transparency containing vessel length, vessel area, and number of junctions (n ​= ​3). (F) A timeline flow chart illustrating the study design. ∗, each group *vs.* 0 group. #, each group *vs.* 0.1 group. $, each group *vs.* 10 group. ∗*p* ​< ​0.05; ∗∗*p* ​< ​0.01; ∗∗∗*p* ​< ​0.001. ^#^*p* ​< ​0.05. ^$^*p* ​< ​0.05.

As one of the most effective ways to salvage a compromised fingertip, the abdominal pocketing procedure is typically performed for patients with vascular compromise that is not resolved with medication [34,35]. Inspired by this procedure, to further improve the repair effect of the TENG, we intend to focus on the individualized TENG, whose pre-vascularization process loaded with VEGF *in vivo,* and clarify the relationship between the pre-vascularization of TENGs and the effect of bridging peripheral nerve defects *in vivo* (Fig. 1F). Similar work as *in situ* prevascularization strategy was reported 3D porous nerve guidance conduits to achieve angiogenesis-mediated neural regeneration [36]. However, *in situ* prevascularization cannot be carried out in the injured location due to the severity of infection and inflammation. If it is carried out in the unaffected limbs, it may cause multiple and even more severe damage of healthy tissues than subcutaneous prevascularization. *In situ* prevascularization is far from the clinical practice. Repair of peripheral nerve injuries using a prevascularized cell-based TENG needed to cost extra three weeks to finish the preparation *in vitro* [11]*.*

The superior vascularized bio-scaffold materials can be screened out by the *in vivo* subcutaneous pre-vascularization tissue engineering technology, providing a basis for further research on the complex interaction between vascularization and its microenvironment. It is also an innovative application of this individualized TENG in peripheral nerve regeneration. The expected results provide an emerging approach and experimental basis for improving the clinical efficacy of repairing long-distance peripheral nerve defects and display its potential for clinical application in the future.

## 2 Results

### 2.1 Subcutaneous prevascularization of VEGF-loaded TENG

Subcutaneous prevascularization model was previously demonstrated suitable for the study of prevascularized bioengineered engraftment [37,38]. In addition, we have shown that the chitosan nerve conduit we used owns a microporous structure that allows blood vessels, nutrients, oxygen, and growth factors to penetrate [32,39]. To determine the optimal condition for subcutaneous prevascularization of the TENG here, we embedded various concentrations (0, 0.1, 1.0, 10.0 ​μg/ml) of VEGF-loaded chitosan nerve conduit and inserted silk fibroin fibers subcutaneously in the back of rats for 4, 7, or 14 days (Fig. 1A). Scanning electronic microscope images showed the appearance of cross section of chitosan conduit, silk fibroin filaments, inner surface and outer surface of chitosan conduit, respectively. Both surfaces of chitosan conduit displayed porous structure (Fig. 1B). Compare with the control group (no VEGF loaded), 1 ​μg/ml VEGF-loaded TENG showed better neovascularization than other groups post-subcutaneous implantation by gross view (Fig. 1C). The embedded TENGs were further tissue-cleared to better explore the prevascularization level. The gross assessments were primarily for superficial neovascularization of TENGs after implantation. 1 ​μg/ml VEGF-loaded TENG displayed best prevascularization grade among groups post-transparency at different time points post-subcutaneous implantation (Fig. 1D). As shown in Fig. 1E, all data of vessel length, vessel area, and number of junctions in the 1 ​μg/ml VEGF-loaded TENG group were significantly higher than in other groups.

### 2.2 Biocompatibility evaluation of VEGF-loaded TENG

**Fig. 2:**
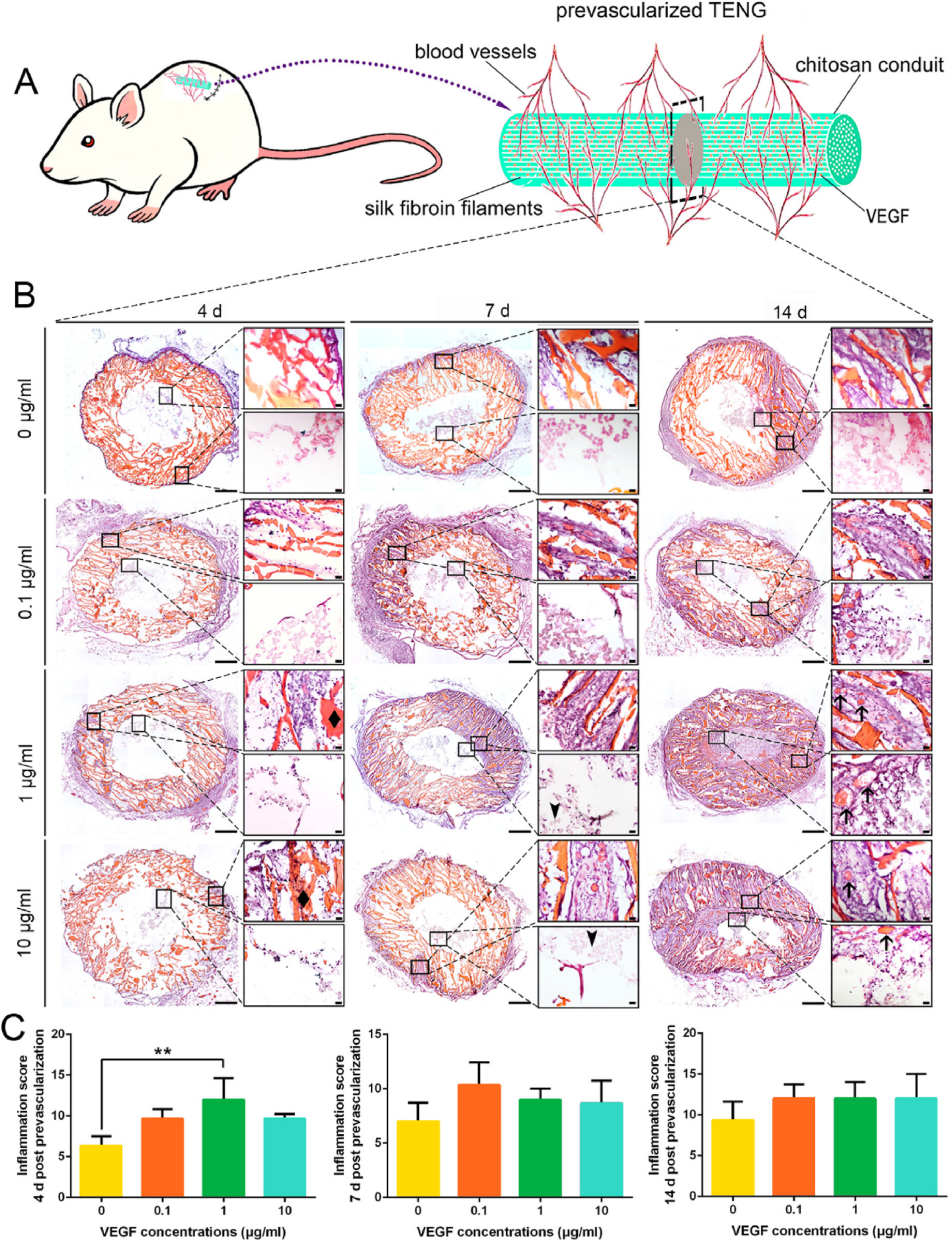
Inflammation score of the prevascularized TENGs after subcutaneous prevascularization. The TENGs with 1 ​μg/ml VEGF caused the fastest penetration of blood vessels from surrounding tissues. More blood vessels grew into the TENGs with VEGF in 1 ​μg/ml concentration at all-time points for prevascularization. (A) A schematic illustration of the prevascularized TENGs. (B) The HE stainings of the middle segments of prevascularized TENGs. The panoramic view and magnified fields of both wall and lumen of the cross-section of nerve conduit were displayed. The arrows indicated the blood vessels in prevascularized TENGs. The arrowheads indicated the silk fibroin filaments. The diamonds indicated the chitosan conduit walls. Scale bar, 500 ​μm and 20 ​μm respectively. (C) Histograms of the inflammation score of prevascularized TENGs at 4 ​d, 7 ​d, 14 ​d post implantation (n ​= ​3). At 4 ​d after prevascularization, the prevascularized TENGs with 1 ​μg/ml VEGF demonstrated higher inflammation state. ∗, each group *vs.* 0 group. ∗∗*p* ​< ​0.01.

When the TENG is implanted *in vivo*, ischemia, inflammation and graft-versus-host reaction will cause accumulation of reactive oxygen species (ROS) at the injury site. The long-term persistent inflammatory responses can aggravate the pathological damage of PNI [40]. To investigate the inflammatory response of various VEGF-loaded TENGs *in vivo*, cryo-cross-section and H&E staining were carried out in the intermediate segment of subcutaneously prevascularized TENGs (Fig. 2A). 1 ​μg/ml VEGF-loaded TENG group caused the fastest penetration of blood vessels from surrounding tissues. And more blood vessels grew into the TENGs at all-time points for prevascularization (Fig. 2B). It displayed a significantly inflammatory status at 4 ​d post-subcutaneous implantation among groups based on the inflammation scores (Fig. 2C). However, because of the much stronger angiogenic capacity in the early stage of 1 ​μg/ml VEGF-loaded TENG, only a statistically significant difference showed in inflammation scores between the 1 ​μg/ml VEGF and control groups. Instead, a high concentration of VEGF (10 ​μg/ml)-loaded TENG group slightly inhibited the inflammatory response. All VEGF-loaded TENG groups showed a similar inflammatory status with the non-VEGF-loaded TENG group 7 ​d and 14 ​d post-subcutaneous implantation. Collectively, these results confirmed 1 ​μg/ml VEGF-loaded TENG may drive the microenvironment to the inflammatory status within early stage (within 4 ​d), and may not affect its long-term biocompatibility post-subcutaneous implantation. Similar to previous studies [41,42], a certain degree of inflammation in early stage (within 7 ​d) contributes to the improvement of the local microenvironment, as myelin debris clearance etc., and further regeneration of peripheral nerves.

### 2.3 Histological analysis of prevascularized TENG

**Fig. 3:**
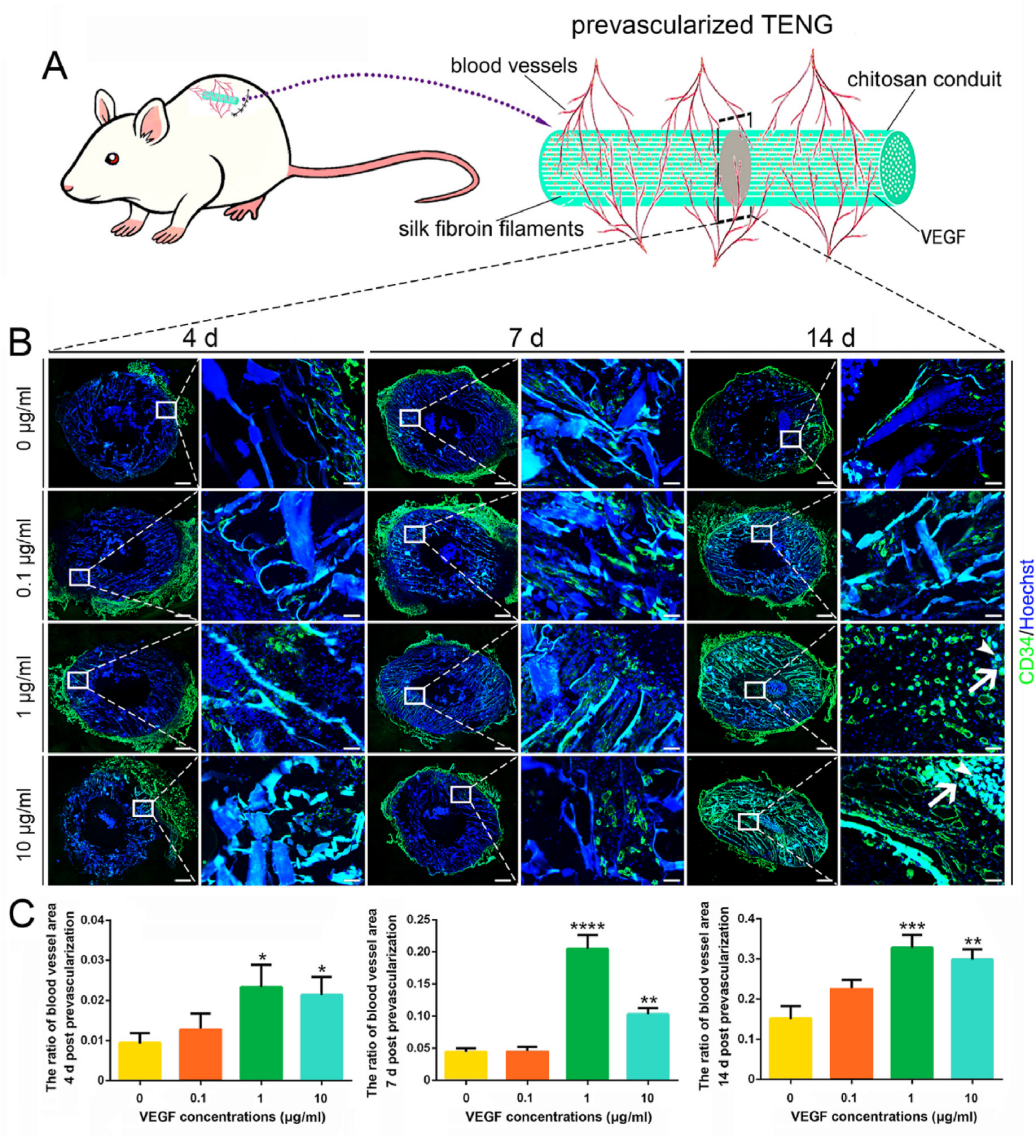
The evaluation of TENGs after subcutaneous prevascularization. The TENGs with 1 ​μg/ml VEGF showed better prevascularization status for out surfaces, walls and inner surfaces of the TENG. It was demonstrated that the silk fibroin scaffolds could guide the VECs migration. (A) A schematic illustration of the prevascularized TENGs. (B) The immunofluorescence stainings of the middle segments of prevascularized TENGs. In addition to the panoramic view, the enlarged local field marked by the rectangular frame was shown. The blood vessels were green. The nuclei were blue. However, the chitosan displayed autofluorescence. The arrowheads indicated the silk fibroin filaments. The arrows indicated the blood vessels along the silk fibroin scaffolds. Scale bar, 500 ​μm and 50 ​μm respectively. (C) Histograms of the ratios of blood vessel areas after prevascularization (n ​= ​3). ∗, each group *vs.* 0 group. ∗*p* ​< ​0.05; ∗∗*p* ​< ​0.01; ∗∗∗*p* ​< ​0.001; ∗∗∗∗*p* ​< ​0.0001. (For interpretation of the references to color in this figure legend, the reader is referred to the Web version of this article.)

To further quantify the prevascularization of VEGF-loaded TENGs, especially the ingrowth of internal blood vessels, CD34 immunofluorescence labeling was performed in the cryo-cross-section of the intermediate segment at 4 ​d, 7 ​d, and 14 ​d post-subcutaneous implantation (Fig. 3A). At 4 ​d, CD34 positive blood vessels grew beyond the exterior wall of TENGs in all groups. By 7 ​d post-subcutaneous implantation, blood vessels distributed around the exterior wall and infiltrated throughout the whole conduit wall of TENGs. Moreover, at 7 ​d, no apparent intraluminal axial blood vessels in the middle segments of TENGs in all groups. At 14 ​d, more CD34-labeled blood vessels were detected throughout the whole conduit wall of TENGs (Fig. 3B). More importantly, numerous axial blood vessels in the TENGs lumens of 1 ​μg/ml and 10 ​μg/ml VEGF groups were painted at 14 ​d, which could directly guide nerve regeneration post nerve bridging. Quantitative analysis suggested that 1 ​μg/ml and 10 ​μg/ml VEGF-loaded TENG groups displayed a significant promotion of prevascularization post-subcutaneous implantation (Fig. 3C). It indicated that subcutaneously prevascularization of VEGF-loaded TENGs needed at least 7 days, while 10 ​μg/ml VEGF-loaded TENG group did not show higher level of prevascularization than 1 ​μg/ml VEGF-loaded TENG group. Thus, this prevascularization model of 1 ​μg/ml VEGF-loaded TENG was deemed appropriate for another bridging sciatic nerve defect model.

### 2.4 Functional analysis of prevascularized TENG

**Fig. 4:**
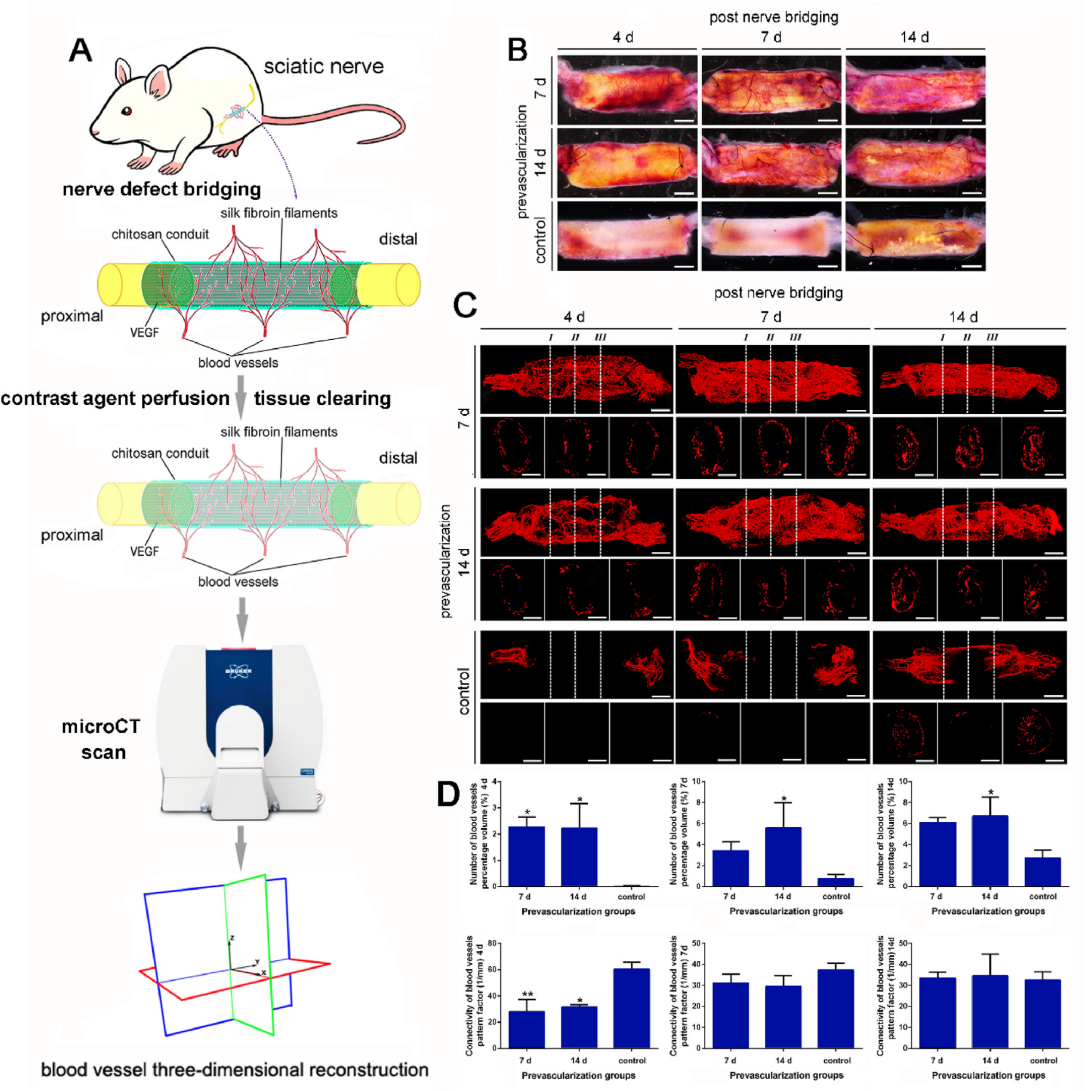
Vascular network anastomosis of the prevascularized TENGs after nerve bridging surgery. The blood perfusion of prevascularized TENGs were rapidly founded by a functional vascular network with both nerve stumps and surrounding tissues. (A) A schematic diagram of the micro -CT scanning and blood vessel three-dimensional reconstruction. (B) The photos of transparented TENGs following prevascularization after nerve bridging at different time points. Scale bar, 2000 ​μm. (C) The blood vessel images by three-dimensional reconstructions at 4 ​d, 7 ​d, and 14 ​d after nerve bridging surgery. The entire functional vascular networks were painted. The cross-sections of three different positions indicated by Ⅰ, Ⅱ, and Ⅲ were also displayed. Scale bar, 2000 ​μm and 1000 ​μm, respectively. (D) Histograms of the vascular network-related parameters (n ​= ​3). The percentage volume (%) represented the number of blood vessels. The pattern factor stood for the connectivity of blood vessels, and a smaller value meant better connectivity. ∗, each group *vs.* 0 group. ∗*p* ​< ​0.05; ∗∗*p* ​< ​0.01.

To investigate whether these blood vessels from prevascularized TENG could rapidly inosculate with the local blood vessels and ultimately bring about functional perfusion, 1 ​μg/ml VEGF prevascularized (7 ​d and 14 ​d) and non-prevascularized TENGs were utilized to bridge 10-mm sciatic nerve in rats, respectively. The contrast agent perfusion, tissue clearing, micro-CT scanning, and blood vessels three-dimensional (3D) reconstruction were applied in all TENGs groups 4 ​d, 7 ​d, and 14 ​d post-nerve bridging (Fig. 4A). The gross views of tissue-cleared TENGs showed the similar vascularization status of prevascularized TENGs that are much better than that in the non-prevascularized TENG group (Fig. 4B). The micro-CT scanning and 3D reconstruction displayed all the perfused blood vessels in both nerve stumps and the TENG segment longitudinally and cross-sectionally (I, II, and III) (Fig. 4C). In 7 ​d and 14 ​d prevascularized TENG groups, the functional vascular networks were rapidly formed 4 ​d post-nerve bridging that is consistent with our hypothesis, prevascularized networks could rapidly inosculate with the neural vascular networks. Both external and internal vascular networks were reconstructed in prevascularized TENG groups (Fig. 4C). No significant differences were detected between 7 d- and 14 d-1 μg/ml VEGF prevascularized TENG groups at 4 ​d, 7 ​d, and 14 ​d post nerve bridging surgery. The regenerative vascular networks were not fully reconstructed in non-prevascularized TENG within 14 ​d post nerve bridging (Fig. 4C). Notably, a functional perfused intraluminal axial vascular network had been rebuilt in prevascularized TENG at 14 ​d after nerve bridging. The number of blood vessels percentage volumes and the connectivity of blood vessels pattern factors in prevascularized TENG groups are much better than in non-prevascularized TENG post nerve bridging surgery (Fig. 4D). The significant differences in vascular connectivity between prevascularized TENGs and control groups at 4 ​d supported a rapid anastomosis of vascular networks after bridging surgery. The time window (4 ​d) of vessels inosculation is consistent with anther previous work [22]. All these indicate prevascularization of TENG contributes to the early reconstruction of local vascular networks.

### 2.5 Prevascularization affect the behaviors of VECs in TENG

**Fig. 5:**
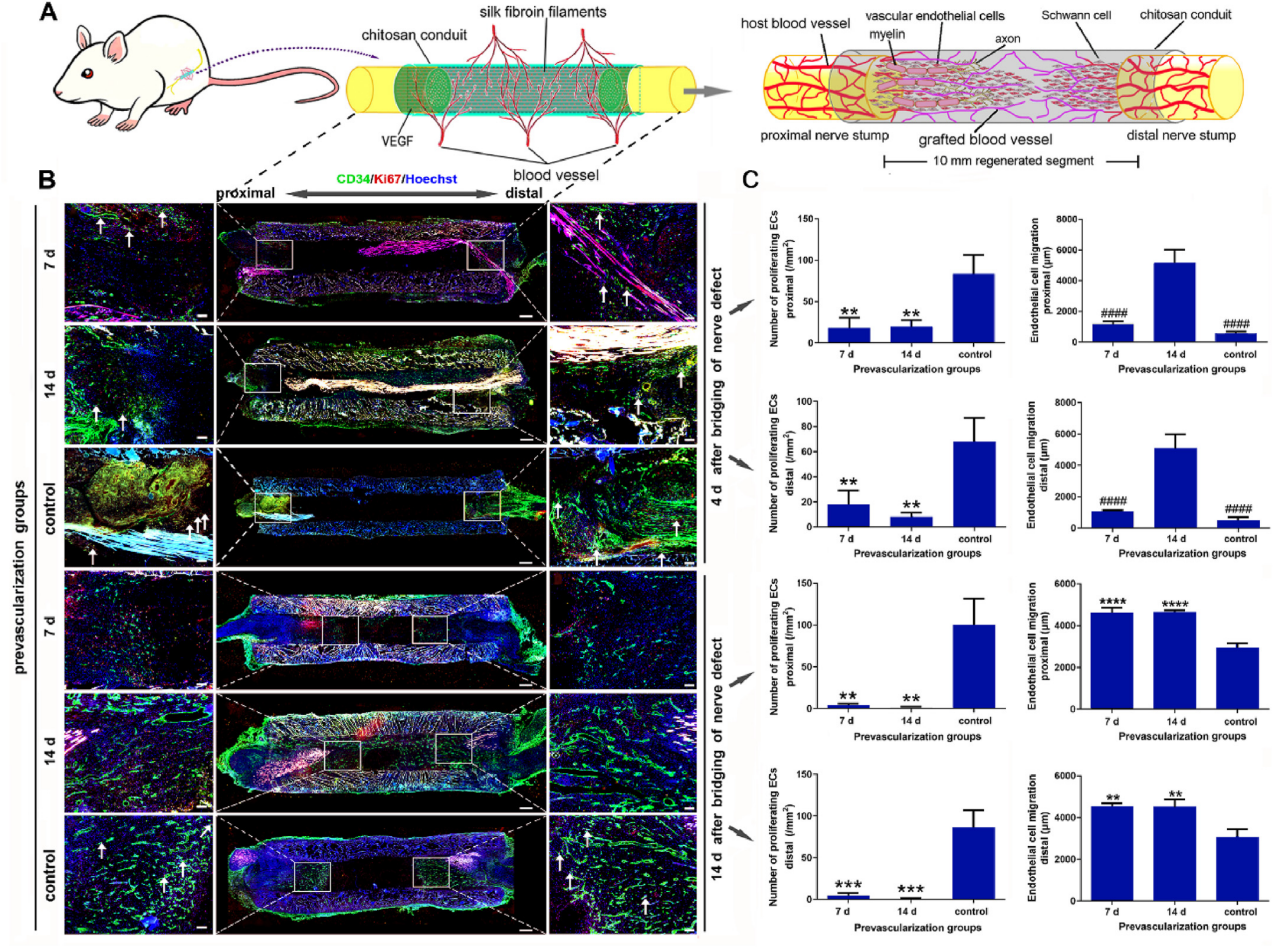
Effects of prevascularization on the VECs after nerve bridging surgery. After 14 ​d of prevascularization, the new blood vessels had filled the conduit lumen. Prevascularization for 7 ​d, compared with the control, pre-grown blood vessels promoted the migration and growth of new vessels after nerve bridging for 14 ​d. Meanwhile, the blood vessels by prevascularization inhibited the proliferation of VECs after nerve bridging because they quickly participate in the reconstruction of functional vascular networks. (A) A schematic diagram of the prevascularized TENGs for nerve defect bridging. (B) The immunofluorescence images of the longitudinal sections of prevascularized TENGs. The magnified fields at both sides of TENGs were marked with rectangular frames. The VECs migration and proliferating VECs were displayed. The blood vessels were green. The proliferating cells were red. The proliferating VECs were co-localized with two positive signals. The nuclei were blue. The arrows indicated the proliferating VECs. Scale bar, 500 ​μm and 100 ​μm respectively. Histograms of the number of proliferating VECs and VEC migration distances were further shown and analyzed (n ​= ​3). ∗, each group vs. control group. ∗∗*p* ​< ​0.01; ∗∗∗*p* ​< ​0.001; ∗∗∗∗*p* ​< ​0.0001. #, each group *vs.* 14 d group. ^####^*p* ​< ​0.0001. (For interpretation of the references to color in this figure legend, the reader is referred to the Web version of this article.)

Vascular integrity is damaged post-PNI and is regulated by VECs via modulating the blood vessel permeability and vascular tone [43]. Rapidly restoring the integrity of blood vessels at the nerve injury site as soon as possible helps promote neural function recovery. To explore whether prevascularization can affect the proliferation and migration of VECs in TENG, 7 d-, 14 d-1 μg/ml VEGF prevascularized and non-prevascularized TENGs were applied to bridge the 10-mm sciatic nerve in rats, respectively. The cryo-longitudinal-section and double immunofluorescence labeling (CD34 and Ki67) were carried out in all TENGs at 4 ​d and 14 ​d post nerve bridging surgery (Fig. 5A). At 4 ​d post nerve bridging surgery, CD34 and Ki67 double immunofluorescence positive blood vessels grew into the intracavity of TENGs in 7 d- and 14 d-1 μg/ml VEGF prevascularized TENG groups. The proliferating VECs in prevascularized TENG groups were significantly less than those in non-prevascularized TENG group. Notable vascularization happened in non-prevascularized TENG group at 14 post nerve bridging surgery, but an unvascularized gap was still existed (Fig. 5B). The implantation of prevascularized TENGs significantly suppressed the proliferation of VECs and significantly promoted migration of VECs in both nerve stumps and TENGs intracavity (Fig. 5C). The proliferation of VECs reflected that prevascularization greatly accelerated mature vascular network reconstruction to relieve early hypoperfusion, so that no more VEC proliferation was required to gradually rebuild functional vascular network. These data indicate that prevascularization of TENG may affect behaviors of VECs to increase the vascular regenerative capacity further.

### 2.6 Prevascularization change the regenerative processes of SCs in TENG

**Fig. 6:**
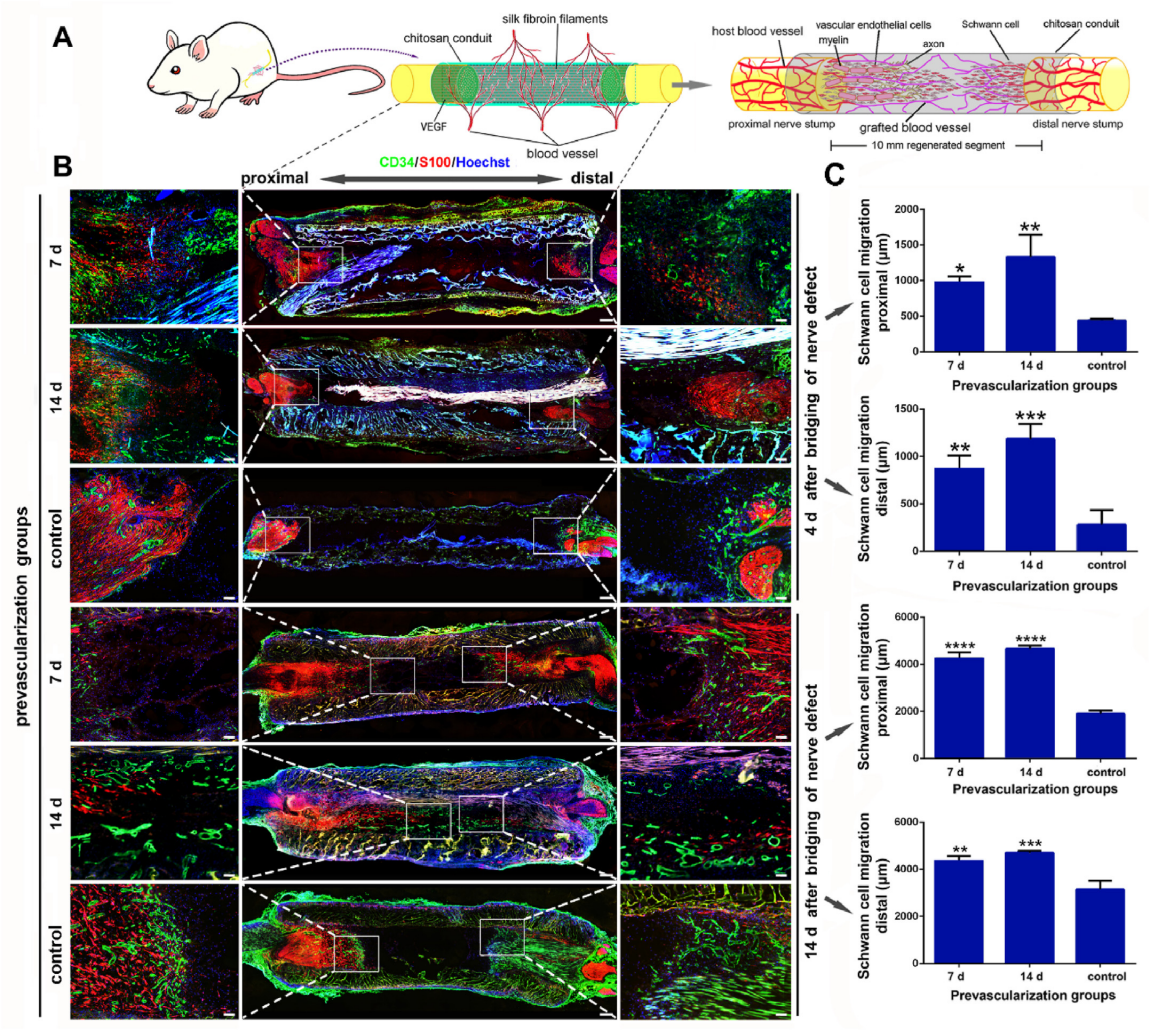
Effects of prevascularization on the SCs migration after nerve bridging surgery. The blood vessels by prevascularization in lumens of TENGs promoted the migration of SCs on both sides of nerve trunks. Compared to the control group, as a guiding bridge, the proximal prevascularized neovascularization significantly accelerated the migration of SCs from the proximal stump. (A) A schematic diagram of the prevascularized TENGs for nerve defect bridging. (B) The immunofluorescence images of the longitudinal sections of prevascularized TENGs. The magnified fields at both sides of TENGs were indicated with rectangular frames. The SCs migration and blood vessels were painted. The blood vessels (CD34 positive, green). The SCs (S100 positive, red). The nuclei (Hoechst 33,342, blue). Scale bar, 500 ​μm and 100 ​μm respectively. Histograms of the SCs migration distances at the proximal and distal stumps of TENGs were counted (n ​= ​3). ∗, each group *vs.* control group. ∗*p* ​< ​0.05; ∗∗*p* ​< ​0.01; ∗∗∗*p* ​< ​0.001; ∗∗∗∗*p* ​< ​0.0001. (For interpretation of the references to color in this figure legend, the reader is referred to the Web version of this article.)

As angiogenesis plays a crucial role in repairing nerve injuries, the crosstalk of VECs and SCs is of particular interest [44]. To discover whether prevascularization can affect the SCs migration in TENG, 7 d-, 14 d-1 μg/ml VEGF prevascularized and non-prevascularized TENGs were applied to bridge the 10-mm sciatic nerve in rats, respectively. The cryo-longitudinal-section and double immunofluorescence labeling (CD34 and S100) were carried out in all TENGs at 4 ​d and 14 ​d post nerve bridging surgery (Fig. 6A). At 4 ​d post nerve bridging surgery, S100 immunofluorescence positive SCs from both nerve stumps migrated longer distance into the intracavity of TENGs than in the non-prevascularized TENG group. Moreover, SCs from both nerve stumps can connect to form the Bünger's band-like structure in 7 d- and 14 d-1 μg/ml VEGF prevascularized TENG groups at 14 ​d post nerve bridging surgery. However, notable SCs migration was not displayed in non-prevascularized TENG group until 14 ​d post nerve bridging surgery (Fig. 6B). The pre-existing blood vessels initiated the migration of SCs in nerve trunks on both sides, that was, into the regeneration stage. However, at 4 ​d after the operation, the control group was still in the preparatory stage before regeneration, and the nerve stumps were more retracted. The implantation of prevascularized TENGs significantly promoted the migration of SCs in both nerve stumps (Fig. 6C). It is different from the classic regenerative process, the Bünger's band formation, in which massive SCs proliferate in distal nerve stump and migrate to the proximal nerve stump. These data indicate that prevascularization of TENG may affect regenerative processes of SCs to further beneficial to neural regeneration.

### 2.7 Prevascularized TENGs promote axonal regrowth

**Fig. 7:**
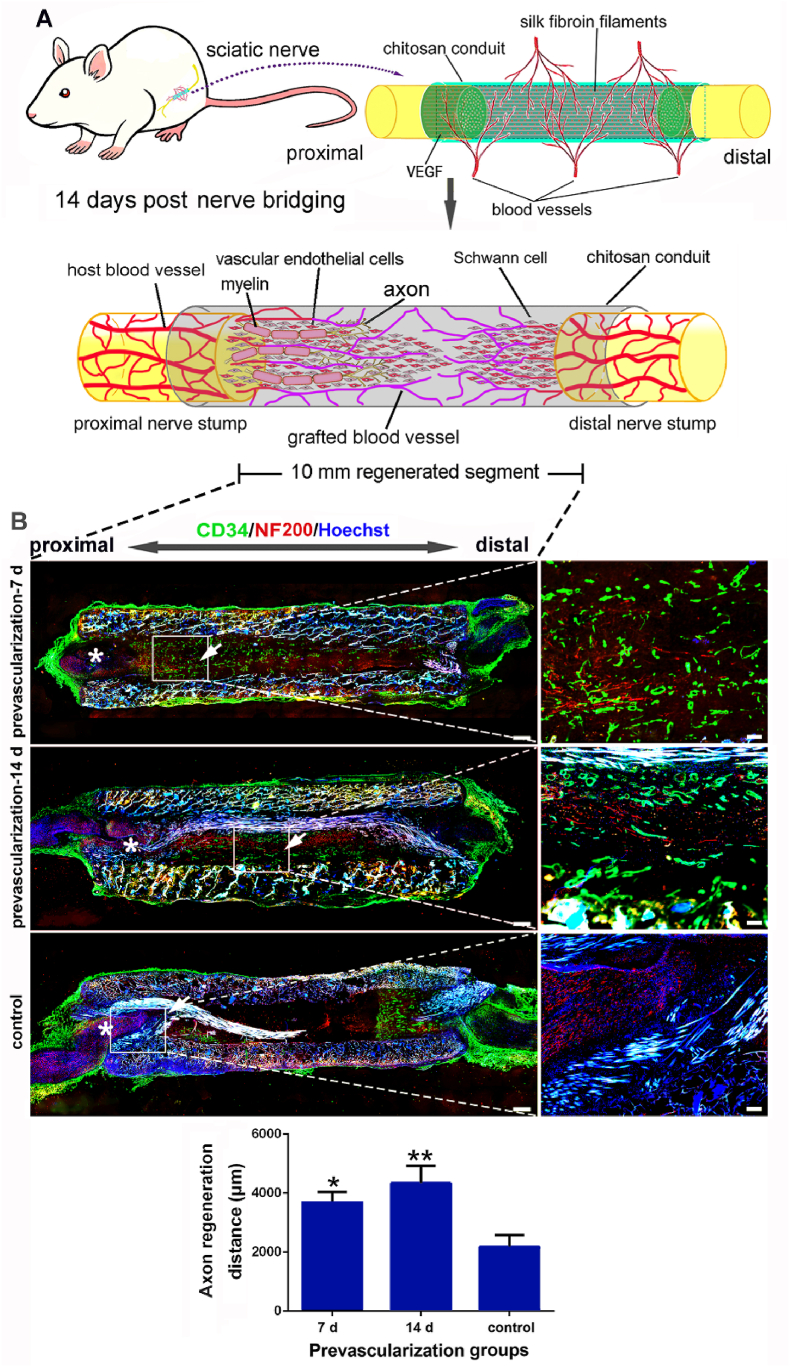
Effects of prevascularization on the axon extension after nerve bridging. Based on the accelerated migration of SCs, the blood vessels of TENGs by prevascularization significantly enhanced the extension and regeneration of axons. (A) A schematic diagram of the prevascularized TENGs for nerve defect bridging. (B) The immunofluorescence images of the longitudinal sections of prevascularized TENGs at 14 ​d after nerve bridging. The magnified fields were indicated with rectangular frames. The axon extension and blood vessels were illustrated. The blood vessels (CD34 positive, green). The axons (NF200 positive, red). The nuclei (Hoechst 33,342, blue). Scale bar, 500 ​μm and 100 ​μm respectively. Histograms of the axon extension distances at the proximal stumps of TENGs were calculated (n ​= ​3). ∗, each group *vs.* control group. ∗*p* ​< ​0.05; ∗∗*p* ​< ​0.01. (For interpretation of the references to color in this figure legend, the reader is referred to the Web version of this article.)

Since the blood vessels may direct SCs migration, interfering the directionality of SCs will lead to inferior axonal regeneration [4]. To examine whether TENG's prevascularization can help axons regrow, 7 d-, 14 d-1 μg/ml VEGF prevascularized and non-prevascularized TENGs were applied to bridge the 10-mm sciatic nerve in rats, respectively. The cryo-longitudinal-section and double immunofluorescence labeling (CD34 and NF200) were carried out in all TENGs at 14 ​d post nerve bridging surgery (Fig. 7A). Under the guidance of VECs and SCs, axonal regrowth was superior in both prevascularized TENG groups to the non-prevascularized TENG group. Axons regenerated twice the distance in prevascularized TENG groups than in non-prevascularized TENG group at 14 ​d post nerve bridging surgery (Fig. 7B). Due to more abundant prevascularized blood vessels at the beginning, the axonal regrowth distance of 14 ​d prevascularization was slightly longer than that of 7 ​d prevascularization. These data indicate that prevascularization of TENGs can promote axonal regeneration by regulating VECs and SCs.

### 2.8 Prevascularized TENGs promote sciatic nerve functional recovery

**Fig. 8:**
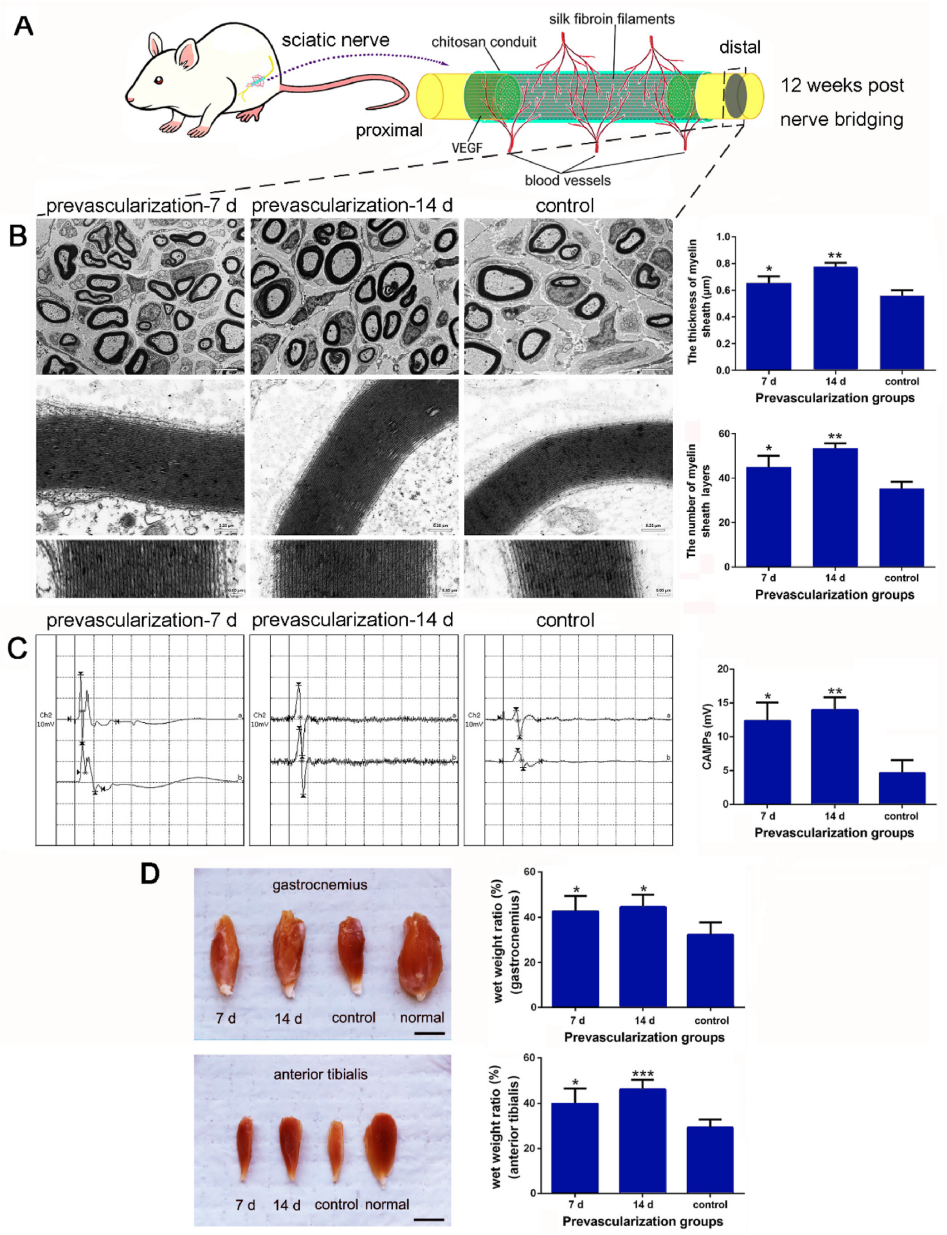
Nerve regeneration assessments of the prevascularized TENGs after nerve bridging. At 12 w after nerve defect bridging, the prevascularization for TENGs reflected a stronger ability to repair nerve defects in regenerated nerve tissue structures, electrical signal conductions, and reinnervation of target organs. (A) A schematic illustration of the prevascularized TENGs and the functional evaluations at 12 w after nerve bridging. (B) The transmission electron microscope pictures of distal regenerated nerves, and histograms of the thickness of myelin sheaths and the number of myelin sheath layers (n ​= ​5). Scale bar, 5 ​μm, 0.25 ​μm, and 0.05 ​μm respectively. ∗, each group vs. control group. ∗*p* ​< ​0.05; ∗∗*p* ​< ​0.01. (C) The electrophysiological images in waveforms and the histograms of CMAPs amplitudes recorded at the proximal end of TENGs (n ​= ​5). “a” meant the records at distal ends. “b” meant the records at proximal ends. ∗, each group *vs.* control group. ∗*p* ​< ​0.05; ∗∗*p* ​< ​0.01. (D) The gross view of dissected gastrocnemius and anterior tibialis on the surgical and contralateral sides. The histograms of the muscle wet weight ratios (%) (n ​= ​5). Scale bar, 1000 ​μm ∗, each group *vs.* control group. ∗*p* ​< ​0.05; ∗∗∗*p* ​< ​0.001.

Based on the prevascularized TENGs roles in proliferation and migration of VECs or SCs, and axonal regeneration early after bridging, we further investigate whether prevascularized TENGs could enhance sciatic nerve functional recovery 12 w post nerve bridging surgery (Fig. 8A). TEM images of the distal nerve segment of cross-sections showed superior myelin sheath thickness and the number of myelin sheath layers in the prevascularized TENG groups than in the non-prevascularized TENG group. No significant difference was noted between various prevascularized TENG groups (Fig. 8B). Moreover, electrophysiological tests were conducted to evaluate the sciatic nerve functional recovery. The compound muscle action potentials (CMAPs) amplitudes were significantly higher in prevascularized TENG groups than in non-prevascularized TENG group. Similarly, no significant difference was found between prevascularized TENG groups (Fig. 8C). The amelioration of muscle atrophy is assessed by the wet weight ratio of the sciatic nerve target muscles, gastrocnemius and anterior tibialis. By 12 w post nerve bridging surgery, both prevascularized TENG groups displayed a superior wet weight ratio of the gastrocnemius and anterior tibialis muscles than that in the non-prevascularized TENG group (Fig. 8D). These data collectively indicate that prevascularized TENGs have the potential to help patients restore peripheral nerve functions.

## 3 Discussion

Vascularization is one of the major challenges hindering the clinical application of tissue engineering products and technologies [45]. Since vascularization plays a crucial role in local circulation and substance exchange, the prevascularization of transplants provides a potential for the successful regeneration of the tissue defect [3]. Even with tremendous progress in tissue engineering biotechnology, the *in vivo* way is still the best to obtain functional vascularization [46]. Unlike the complex and diverse *in vitro* prevascularized tissue engineering technology [6,11], the *in vivo* prevascularized tissue engineering technology has been applied to some basic researches [[47], [48], [49], [50]]. The main advantages of prevascularization *in vivo* are the autologous microenvironment, which can avoid the exposure to immunogenic products or unfavorable biocues *in vitro* [51].

Prior to our work, to establish immediate blood perfusion of the graft after repairing surgery, as one of the strategies, the flap technique was applied to surgically suture the blood vessels from the graft to the host tissue. The transplantation of a graft into a muscle flap was carried out to finish the prevascularization. The newly formed and the host blood vessels were surgically anastomosed when the prevascularized graft was transferred to the target site. However, this strategy owns several disadvantages as tissue loss, the selections of materials and growth factors doses [52], high requirement of micro-surgical technique. Emerging evidence indicates that an *in vitro* cellular TENG (fibroblasts, SCs and VECs seeded) may promote rapid inosculation with the blood vessel networks in the host after grafting [11]. However, the construction of this TENG needs over 40 days, and the capillary-like structures of prevascularized TENG connected to the host blood vessel networks 14 more days after grafting. Establishing of an efficient and easy-to-follow TENG prevascularization method is of great significance for the extensive clinical application of TENGs in the treatment of peripheral nerve injuries.

The design of the present TENG based on our previous work and experience over past two decades. The neural conduit could guide the direction of peripheral nerve regeneration and prevent fibroblasts or local inflammatory cells aggregated excessively. The micropore structure of chitosan neural conduit help to promote the vascularization level of TENG. More vessels could pass through the wall of neural conduit to ameliorate the local microenvironment for neuroregeneration. The silk fibroin filaments play crucial roles in directing the vascular, axonal, and myelin regeneration [30,33,53,54].

The present work clarified the optimized concentration of VEGF loaded in the TENG and the time of cutaneous prevascularization. Interestingly, the functional vascular networks were rapidly reconstructed 4 ​d post-nerve bridging in prevascularized TENG group. Based on out data shown in Fig. 1C–E, 1 μg/ml VEGF-loaded TENG displayed best prevascularization grade among groups at different time points post-subcutaneous implantation. Increasing the VEGF dose induced a gradual increase in EC proliferation (Fig. 1A). Although VEGF is dose-dependent for endothelial cell proliferation, the cell proliferation rate will reach a plateau when the concentration of VEGF reaches a certain high level [55]. In accordance with the previous work, 1 ​μg/ml VEGF-loaded TENG displayed best prevascularization grade among groups post-transparency at different time points post-subcutaneous implantation (Fig. 1D). As shown in Fig. 1E, all data of vessel length, vessel area, and number of junctions in the 1 ​μg/ml VEGF-loaded TENG group were significantly higher than in other groups in the present work. The 10 ​μg/ml VEGF-loaded TENG group did not show higher level of prevascularization than 1 ​μg/ml VEGF-loaded TENG group.

The blood vessels dissected once the prevascularized-TENG was harvested from the subcutaneous pouch reconnected (vascular inosculation) to the host blood vessels from the nerve injury site post nerve-bridging surgery. These inosculated blood vessels are proved functional by a series of steps as contrast agent perfusion, tissue clearing, microCT scan, and blood vessels 3D reconstruction. The efficiency of TENG vascularization is sufficient for clinical translation in the future. The microvascular vessels would further pruned and remodeled according to the needs of peripheral nerve regeneration [56]. The data in Fig. 4C & D prove above description and no significant difference in the recovery of nerve function between the two groups post sciatic nerve bridging surgery (Fig. 7). These indicated that the level of vascularization and the restoration of neurological function are only proportional to a certain range. Superior prevascularization of TENG did not fully represented better recovery of nerve function.

To obtain more mature vascular networks in TENG, we extended the prevascularization period to 7 ​d and 14 ​d for the behavioral analysis of VECs and SCs post nerve bridging surgery, respectively. The prevascularization of TENG significantly suppresses the proliferation of VECs, and encourages the bidirectional migration of VECs and SCs in the TENG. The SCs from both proximal and distal nerve stumps migrate into the nerve bridge and form SC cords, which is crucial to successful peripheral nerve repair following transection injury [6]. The bands of Büngner are well-known aligned tubular guidance structures that play essential roles in driving axonal regeneration, which typically forms in distal nerve stumps. If the nerve defect is too large, SCs commonly fail to infiltrate the TENGs and connect both nerve stumps, resulting in obviously inferior functional recovery [57]. The atrophic SCs in the distal nerve stump lead to reduced neurotrophic growth factors (NGF) and hamper axonal extension. The atrophic and denervated target muscles limit the functional recovery even if axons may regenerate into the target muscles [2]. In our work, prevascularization of TENGs could activate bidirectional SCs migration and connect both nerve stumps within 14 ​d. It is superior to the typical bands of Büngner formation.

The biomaterial-based approaches may separately impact both spatial and temporal neovascularization. The combination of spatio-temporal strategies will play synergistic roles in accelerating vascularization. The angiogenic peptides functionalized biomaterials have been proved by promoting adhesion, cell migration, proliferation, and organization [58]. The complexity and controllability must be fully considered to construct vascularized grafts that are sufficiently intricate to mimic the natural blood vessel structures *in vivo* [8].

As commonly worried about, three surgical interventions, supposed to be a limitation of this work, are required in prevascularized TENGs application, i.e., cutaneous implantation of the TENG for prevascularization firstly, the surgical removal of the prevascularized TENGs secondly and the implantation to bridge the nerve defect lastly. However, it's impossible to finish the treatment of clinical peripheral nerve defects by a single surgery. The secondary-stage or third-stage surgery is usually required to repair the nerve defects gradually. The TENG can be transplanted subcutaneously to complete prevascularization during the debridement. Based on the present work, the transplantation of prevascularized TENG can be completed in the second stage of nerve repair. Although applying an *in situ* prevascularization strategy to achieve angiogenesis-mediated neural regeneration was reported [6], the subcutaneous prevascularization of TENG is much easier, safer, and less traumatic than that by *in situ* prevascularization to the patients.

Another limitation of the present work is that although VEGF has a significant effect on promoting vascular growth, it is inherently unstable when directly used *in vivo*. The local inflammatory microenvironment after injury will exacerbate this instability [59]. Applying an effective drug release system will help VEGF keep stable and continuous releasing to perform superior bioactive roles.

## 4 Conclusion

In summary, our findings have clarified that an individualized TENG consisting of VEGF induced host vascular network, chitosan nerve conduit, and inserted silk fibroin fibers obtain superior repairing effect than that of the non-prevascularized control group in rats suffering 10 ​mm sciatic nerve defect injury. The early vascularization of TENG drives axonal regrowth by facilitating bidirectional SCs migration that is various to the classic SCs regenerative procession, the bands of Büngner formation. This pre-vascularized TENG can effectively increase remyelination, enhance electrophysiological functional recovery, and prevent atrophy of the target muscles. The present work's optimal prevascularized conditions and time window may be generalizable to other tissue engineering fields. This neural tissue-engineered pre-vascularization technique provides a potential approach to discover individualized TENG and explore the innovative neural regenerative process.

## 5 Materials and methods

### 5.1 Prevascularized TENGs

Rats were deeply anesthetized by an intraperitoneal injection of compound anesthetic (chloral hydrate 4.25 ​g, magnesium sulfate 2.12 ​g, sodium pentobarbital 886 ​mg, ethanol 14.25 ​ml, and propylene glycol 33.8 ​ml in 100 ​ml) [60]. The TENG is consist of chitosan neural conduit (i.d. 2.0 ​mm) inserted with about 120 silk fibroin fibers (1.4 ​cm long, diameter 8 ​μm) were soaked in the different concentrations of recombinant human VEGF protein (R&D) aqueous solutions for 24 ​h. The chitosan neural conduit was prepared as described previously (please refer to Chinese patent ZL 0110820.9 and a published work [61] for technical details). The SF fibrous fillers were prepared as described previously [53]. Two TENG combined with VEGF were implanted subcutaneously on both sides of the midline of the back of rat, one of which was selected to repair its own sciatic nerve injury. The linear distance between the two grafts on each side was about 2 ​cm. The time for prevascularization was 4 ​d, 7 ​d and 14 ​d. We further dissected the fibrous capsule at both ends of the conduit post subcutaneously prevascularization. Then, both nerve stumps will be inserted into the prevascularizd TENG. Although this resulted in localized microvascular destruction, it also caused vascular inosculation between the graft microvascular network and the host's regenerated microvascular network according to our data shown in Fig. 4C.

### 5.2 Sciatic nerve defect surgery

All animals were deeply anesthetized. The skin and muscle were incised to expose the sciatic nerve at the left mid-thigh. An 8-mm segment of the sciatic nerve was resected to form a 10 ​mm gap following slight retraction of the nerve stumps. We further dissected the fibrous capsule at both ends of the TENG post subcutaneously prevascularization. The sciatic nerve defects were bridged by chitosan neural conduits (control) and prevascularized TENGs. The nerve stumps on both sides were inserted into the lumen of 12 ​mm long TENG about 1 ​mm. Finally, the muscle layers and skins were closed with sutures. The animals were housed in a temperature-controlled environment and allowed food and water ad libitum. Adult female Sprague-Dawley (SD) rats about 200 ​g were acquired from the Experimental Animal Center of Nantong University (License No. SYXK (Su) 2017–0046). All experimental protocols were approved by the Administration Committee of Experimental Animals, Jiangsu Province, China, in accordance with the guidelines of the Institutional Animal Care and Use Committee, Nantong University, China (Inspection No: 20190225–004).

### 5.3 Electromyogram

Under deeply anesthesia, the surgical site at the left mid-thigh level was reopened and the sciatic nerve was re-exposed. Electrical stimuli were applied to the sciatic nerve trunk at the distal and proximal ends of the graft respectively. Compound muscle action potentials (CMAPs) were recorded on the gastrocnemius belly. The assessments of normal sciatic nerve CMAPs were conducted at the uninjured contralateral side.

### 5.4 Blood vessel three-dimensional reconstruction and analysis

Animals were deeply anesthetized. Rats were infused with about 500 ​mL NS mixed with 0.8 ​mL heparin sodium (Changzhou Qianhong Pharmaceutical Co., Ltd, Changzhou, China) in a final concentration of 10 U/mL via the left ventricle. Then blue Microfil compounds (Flow Tech, Inc., Carver, Massachusetts, USA) were infused with a 50 ​mL syringe through the aorta. The perfusion was not ended until the contrast agents outflow from the right atrium [62]. After the curing of Microfil compounds, the prevascularized TENGs repairing the defects were collected by a careful dissection and performed with Micro-CT scanning. The samples were scanned by SkyScan1172 Micro-CT (Bruker Corporation, Billerica, USA) under conditions of voltage 40 ​kV, current 250 ​μA and resolution 7.96 ​μm. And the images of three-dimensional blood vessels were reconstructed, and vascular parameters including number, size and connectivity were analyzed in a unified manner by the software SkyScan CTVOX 2.1 (Fig. 4C and D). the full-scale blood vessels of the transparented scaffold pictures (Fig. 1D and E) were analyzed quantitatively using Angio Tool software (https://ccrod.cancer.gov/confluence/display/ROB2/Home). The ratio of blood vessels area was analyzed by Image J software (Fig. 3B and C).

### 5.5 Tissue sample processing

Rats were anesthetized and perfused with normal saline (NS) and 4% paraformaldehyde via the ventricle. The prevascularized TENGs implanted subcutaneously and the bridge segment including nerve stumps on both sides were harvested. The bilateral gastrocnemius and anterior tibialis were removed and measured to calculate the wet weight ratio. The photos of implanted prevascularized TENGs and muscles were taken with a digital camera (EOS77D, Canon). The collected tissues were post-fixed for 6–8 ​h at 4 ​°C, frozen and cut into 12-μm-thick slices. The prevascularized TENGs both implanted and bridged were firstly transparented in gradient glycerol aqueous solutions from 50%, 75%, 85%–100% successively for 24 ​h, and photographed under the stereomicroscope (AZ100, Nikon). The frozen sections were then stained with hematoxylin-eosin (HE) and immunofluorescence staining to analyze angiogenesis, inflammation or nerve regeneration.

### 5.6 Inflammation score

The inflammation degrees of the prevascularized TENGs immersed with different VEGF concentrations were semi-quantitatively scored following HE staining of the cross sections (ISO 10993–6: 2007) [28] calculated by vascularization, infiltration of inflammatory cells including polymorphonuclear leukocytes, lymphocytes, plasma cells, macrophages and giant cells [60,63,64]. The assessment was conducted by two professional pathologists in a double-blind manner. The 3 random fields with high magnification ( ​× ​400) were used for inflammation evaluation. Images of HE staining were photographed under upright microscopy (AxioImager M2, Zeiss).

### 5.7 Immunofluorescence staining

The tissue slices were blocked with 5% goat serum for 1 ​h at 37 ​°C, incubated with primary antibodies overnight at 4 ​°C, and then secondary antibodies for 1 ​h at room temperature. Primary antibodies included goat anti-CD34 antibody (1:50 dilution, R&D), rabbit anti-S100 antibody (1:200 dilution, Abcam), rabbit anti-Ki67 antibody (1:200 dilution, Sigma), mouse anti-NF200 antibody (1:200 dilution, Sigma). Secondary antibodies included donkey anti-goat IgG-Alex-488 (1:500 dilution, Abcam), sheep anti-rabbit IgG-Cy3 (1:1000 dilution, Abcam) and donkey anti-mouse IgG-Alex-594 (1:800 dilution, Invitrogen). Nucleus was marked using Hoechst 33,342 (1:5000 dilution, Life Technologies). Images were acquired under a fluorescence microscopy (AxioImager M2, Zeiss).

### 5.8 Scanning electron microscope

To characterize the chitosan conduit and silk fibroin filaments using scanning electron microscopy (SEM), the samples were fixed in 4% glutaraldehyde and postfixed with 1% OsO_4_, dehydrated in a graded series of ethanol, which were replaced by *tert*-butyl alcohol. Afterward, samples processed were dried in a freeze drier (Hitachi, ES-2030, Japan) and coated with platinum using a JEOL JFC-110E Ion Sputter, followed by observation under a Philips XL-30 scanning electron microscope (Eindhoven, the Netherlands).

### 5.9 Transmission electron microscope

The distal nerve trunk of bridge was collected, post-fixed in 4% glutaraldehyde, and embedded in Epon 812 epoxy resin (Sigma) [28]. Ultrathin sections were conducted and stained with lead citrate and uranyl acetate. The morphology of regenerating nerves was observed under a transmission electron microscope (JEOL Ltd., Tokyo, Japan). The 3 random fields with low magnification per animal were used for myelin sheath thickness statistics. The 5 random fields with high magnification per animal were selected for myelin sheath layers count.

### 5.10 Statistical analysis

The data were presented as means ​± ​standard deviation (SD). One-way analysis of variance (ANOVA) was used for multiple comparisons among groups. Statistical analysis was performed by using Graph-Pad Prism 6.0 software (GraphPad Software Inc., La Jolla, CA, USA). A *p*-value< 0.05 was considered as statistically significant.

## Ethics statement

Adult female Sprague-Dawley (SD) rats about 200 ​g were acquired from the Experimental Animal Center of Nantong University (License No. SYXK (Su) 2017–0046). All experimental protocols were approved by the Administration Committee of Experimental Animals, Jiangsu Province, China, in accordance with the guidelines of the Institutional Animal Care and Use Committee, Nantong University, China (Inspection No: 20190225–004).

## Credit author statement

C·B.X. and H.Z. conceived the research, supervised the project and provided research direction, including all experimental designs. T.M.Q. and M.Y.L. designed and prepared the chitosan neural scaffold and silk fibroin filaments. H.K.W., P.Z., P.J.L., X.X., G.W. and X.D.C. performed the *in vivo* experiments. H.K.W. and P.Z. carried out the micro-CT scanning and data analysis. Y.L. provided pathological data analysis. H.K.W., H.Z., T.Y.H. and P.Z. verified data integrity and performed the statistical analyses. H.K.W., C·B.X., and H.Z. interpreted the data and co-wrote the manuscript. All the authors reviewed the manuscript.

## Declaration of competing interest

The authors declare that they have no known competing financial interests or personal relationships that could have appeared to influence the work reported in this paper.

## Data Availability

Data will be made available on request.
